# COVID Impact and Response in Rural Age-Friendly Communities

**DOI:** 10.1093/geroni/igaa057.3433

**Published:** 2020-12-16

**Authors:** Patricia Oh, David Wihry, Jennifer Crittenden

**Affiliations:** 1 AARP, Bowdoinham, Maine, United States; 2 University of Maine, Bangor, Maine, United States

## Abstract

Maine is an age-friendly state with 72 communities enrolled in the AARP Network of Age-Friendly States and Communities and another 25+ using a similar approach to engage residents and local organizations in community planning and development that enhances the health and well-being of older people. The majority (83%, n=60) of Maine’s age-friendly communities have fewer than 10,000 residents and only two have populations over 20,000. Before the pandemic, rural Mainers struggled to find safe and convenient transportation options, afford healthy food, access primary and secondary medical care, and participate in activities to stay physically fit and socially connected. Age-friendly community efforts attempted to address those issues by building on community strengths and assets to develop local solutions. The challenges of rural aging were amplified by the pandemic and reliance on older community leaders and volunteers, who were seen as an at-risk population and thus strongly encouraged to avoid social contact. This study offers a narrative exploration of how rural age-friendly communities responded to COVID-19. The leaders of 71 age-friendly communities were contacted by phone, email, or Zoom between May 15 and August 30. Thirty-nine of the communities (54.9%) actively partnered with residents and organizations to address needs during the pandemic. Identified themes described the challenges faced in developing an effective response and new opportunities to expand the age-friendly work in the community. Suggestions for developing new partnerships between age-friendly initiatives and local organizations were highlighted. A key practice implication is the vital importance of intergenerational collaborations in rural areas.

